# Low PR in ER(+)/HER2(−) breast cancer: high rates of *TP53* mutation and high SUV

**DOI:** 10.1530/ERC-18-0281

**Published:** 2018-11-08

**Authors:** Sung Gwe Ahn, Chang Ik Yoon, Jae Hoon Lee, Hye Sun Lee, So Eun Park, Yoon Jin Cha, Chihwan Cha, Soong June Bae, Kyung-A Lee, Joon Jeong

**Affiliations:** 1 Department of Surgery, Gangnam Severance Hospital, Yonsei University College of Medicine, Seoul, Republic of Korea; 2 Department of Nuclear Medicine, Gangnam Severance Hospital, Yonsei University College of Medicine, Seoul, Republic of Korea; 3 Biostatistics Collaboration Unit, Yonsei University College of Medicine, Seoul, Republic of Korea; 4 Department of Pathology, Gangnam Severance Hospital, Yonsei University College of Medicine, Seoul, Republic of Korea; 5 Department of Laboratory Medicine, Gangnam Severance Hospital, Yonsei University College of Medicine, Seoul, Republic of Korea

**Keywords:** progesterone receptor, *TP53* mutation, SUV, breast cancer

## Abstract

On the basis of *TP53* mutations and standardized uptake values (SUVs) from 18F-fluorodeoxyglucose positron emission tomography (18F-FDG-PET), we sought to enhance our knowledge of the biology underlying low progesterone receptor (PR) expression in estrogen receptor (ER)-positive/human epidermal growth factor receptor-2 (HER2)-negative tumors. This study included 272 patients surgically treated for ER-positive, HER2-negative breast cancer and who had undergone *TP53* gene sequencing. Of these, 229 patients also underwent 18F-FDG PET or PET/CT. Mutational analysis of exons 5–9 of the *TP53* gene was conducted using PCR amplification and direct sequencing. The SUVs were measured using 18F-FDG-PET scan images. Twenty-eight (10.3%) tumors had a somatic *TP53* mutation. The *TP53* mutation rate was significantly higher in low-PR tumors than in high-PR tumors (17.1% vs 7.9%, *P* = 0.039). Low-PR tumors had significantly higher median SUVs than high-PR tumors (*P* = 0.046). The multivariable analysis revealed that SUV and age remained independent variables associated with low PR expression. An adverse impact of low PR expression on recurrence-free survival was observed in the multivariable Cox regression hazard model. We provide clinical evidence that genetic alteration of the *TP53* gene and dysregulated glucose metabolism partly involve low PR expression in ER-positive and HER2-negative breast cancer.

## Background

In estrogen receptor (ER)-positive breast cancer, progesterone receptor (PR) expression is generally considered a marker of an intact estrogen-responsive pathway (Horwitz & McGuire 1975). In addition, patients with ER-positive/PR-negative breast cancers have a worse prognosis than that of patients with PR-positive tumors (Clark* et al*. 1983). Recently, two studies suggested that low or negative PR expression can be used to identify ‘luminal B-like’ cancer, which has a poor outcome and is distinguished from ‘luminal A-like’ cancer in ER-positive/human epidermal growth factor receptor-2 (HER2)-negative cancer (Cancello* et al*. 2013, Prat* et al*. 2013).

Therefore, many investigators have tried to dissect tumor biology regarding the loss of PR in ER-positive cancer. Previous studies using human tumor samples suggested several lines of evidence that a higher rate of *TP53* mutation (Olivier* et al*. 2006), DNA copy-number gain or increasing PI3K/mTOR gene signature (Creighton* et al*. 2009), and activation of the growth factor signal pathway (Arpino* et al*. 2005) could be associated with loss of PR expression in ER-positive cancer. However, these studies included both ER-positive/HER2-positive and -negative tumors; thus, previous findings on the loss of PR expression might be affected by HER2 overexpression. Molecular studies based on next-generation sequencing showed that HER2-enriched tumors more frequently harbor *TP53* mutation than luminal A or luminal B tumors (Desmedt* et al*. 2012, Bertheau* et al*. 2013). Furthermore, standard of care for ER-positive/HER2-positive patients is anti-HER2 therapy plus chemotherapy followed by endocrine therapy, differing from that for ER-positive/HER2-negative patients (Goldhirsch* et al*. 2013).

Our group previously reported the prognostic influence of the standardized uptake value (SUV) on 18F-fluorodeoxyglucose positron emission tomography (18F-FDG-PET), which represents glucose uptake, in ER-positive breast cancer (Ahn* et al*. 2014*a*,*b*). In addition, we found a positive correlation between the SUV and the 21-gene recurrence score (RS) in ER-positive/HER2-negative disease (Ahn* et al*. 2017).

In this study, we first questioned whether the rate of *TP53* mutation was higher in low-PR tumors than that in high-PR tumors among ER-positive/HER2-negative breast cancer. Second, we compared SUVs according to PR status in these tumors. This study sought to enhance our understanding of the biology underlying low PR expression within ER-positive/HER2-negative tumors.

## Patients and methods

### Patients

This study included 272 patients surgically treated for ER-positive, HER2-negative breast cancer and who had undergone *TP53* gene sequencing between March 2007 and December 2015 at Gangnam Severance Hospital, Yonsei University College of Medicine. Patients who were treated with neoadjuvant chemotherapy and those who were diagnosed with recurrent or metachronous breast cancer were excluded from this study. Patients with ductal carcinoma *in situ* were also excluded. Of the 272 patients, 229 also underwent 18F-FDG PET or PET/computed tomography (CT) as part of routine preoperative staging.

The staging was performed according to the 7th edition of the American Joint Committee on Cancer system (Edge & Compton 2010). The Elston-Ellis modification of the Scarff-Bloom-Richardson grading system was used for histologic grading. Adjuvant systemic therapy and/or radiotherapy were administered according to the standard guidelines based on patient age, primary tumor characteristics and axillary lymph node status. Endocrine therapy was administered to all patients. The follow-up protocol included planned regular visits every 6 months; missed appointments were followed-up by telephone calls to minimize the number of patients lost to follow-up and to improve the accuracy of the survival data. The final update to the clinical database was made in March 2018. Our study was approved by the Institutional Review Board of Gangnam Severance Hospital, Yonsei University, Seoul, Korea, in accordance with the good clinical practice guidelines under the Declaration of Helsinki.

### Definition of low PR expression and immunohistochemical (IHC) study

We defined low PR expression based on the modified Allred system (Harvey* et al*. 1999): low, Allred score 0–4; and high, Allred score 5–8. ER expression was also evaluated in the same manner. All tumors included in this study were ER positive (Allred scores ≥3). HER2 status was evaluated according to the 2013. American Society of Clinical Oncology/College of American Pathologists (ASCO-CAP) guidelines (Wolff* et al*. 2013) and was negative for all cases. The antibodies used for the IHC study were described previously. Ki67 expression was measured by an experienced pathologist and reported as a percentage score (range 0–100%) of positive tumor cells.

### *TP53* Sanger sequencing

Mutational analysis of exons 5–9 of the *TP53* gene was conducted using PCR amplification and direct sequencing (Kim* et al*. 2014). The primers designed to amplify the exons and flanking introns of the *TP53* gene were described previously (Kim *et al*. 2014). Briefly, PCR was performed using an Accu-PowerTM Premix (Bioneer, Daejeon, Korea) under the following amplification conditions: 94°C for 4 min followed by 50 cycles of 94°C for 1 min, 60°C for 30 s and 72°C for 30 s and final extension at 72°C for 15 min. Purified PCR products obtained using a QIAquick Gel Extraction kit (Qiagen) were used for sequencing with a Big Dye Terminator Cycle Sequencing Ready Reaction kit (Applied Biosystems). The thermal cycler conditions were as follows: 96°C for 5 min followed by 24 cycles of 96°C for 10 s, 50°C for 5 s and 60°C for 4 min and final extension at 72°C for 5 min. The sequences were analyzed using an ABI 3500Dx system (Applied Biosystems). The *TP53* sequences were compared to the GenBank database sequence (accession number NC_000017.9) (Cancer Genome Atlas Network 2012). Both forward and reverse strands were sequenced to confirm the full sequence length (bp) and nucleotide alterations (Kim* et al*. 2013).

### 18F-FDG PET or PET/CT method

The procedure for 18F-FDG PET or PET/CT was as previously reported (Ahn *et al*. 2014*b*, Lee* et al*. 2016). The SUV was calculated by measuring the 18F-FDG uptake by the primary tumor in the region of interest, as follows: SUV = (maximal radioactivity concentration in the region of interest)/(injected dose/patient’s weight (kg)).

### Statistical analysis

Continuous variables were compared using Mann–Whitney *U* test. Discrete variables were compared using *χ* ^2^ or Fisher’s exact tests. The SUV was incorporated into the analyses as a continuous variable. Kolmogorov–Smirnov tests were used to assess the normal distribution of the SUV. To identify predictive factors for low PR expression, the binary logistic regression analysis was performed using all variables. The variables with *P* value <0.05 were included in the full multivariable model, and the stepwise backward Wald method was used to arrive at the final model. No adjustments were made for multiple statistical testing.

The recurrence-free survival time (RFS) was measured from the date of the first curative surgery to the date of the first tumor recurrence, including loco-regional recurrence, distant metastasis or death. The Kaplan–Meier method was used to estimate the RFS, and the estimated survival curves were compared using the log-rank test. The Cox’s regression-hazard model was used for univariable and multivariable survival analyses. Significant variables in univariable analysis are included in the multivariable model. Variables that were significant at the 0.05 level were then selected to build a predictive multivariable Cox regression survival model. A backwards selection method (*P* = .05 for inclusion and *P* = .05 for exclusion, wald method) was used to identify significant prognostic factors for the RFS. In addition, a predictive ability of multivariable was measured using Harrell c-statistics (Harrell* et al*. 1996) and the concordance index (c-index) was calculated to measure the concordance for time-to-event data, in which increasing values between 0.5 and 1.0 indicated improved prediction. PASW Statistics for Windows, version 18.0 (SPSS, Inc.) and the R software (version R-3.3.3; https://www.r-project.org) were used to perform these analyses. Statistical significance was defined as *P* values <0.05 or 95% CIs.

## Results

### Baseline characteristics

Two hundred seventy-two patients with ER-positive, HER2-negative tumors were included in the analyses. Two hundred two (74.3%) and seventy (25.7%) patients had a high and low PR expression, respectively, while 241 (88.6%) and 31 (11.4%) patients had high and low ER expression, respectively. The baseline characteristics were compared according to PR expression (Table 1). The median age of the low PR group was significantly younger than that of the high PR group (*P* < 0.001). There were no differences in anatomical staging, whereas histologic grade tended to be higher in low-PR tumors (*P* = 0.064). Also, the proportion of tumors with ER expression differed significantly according to PR expression (*P* = 0.019), while the proportion of tumors with high Ki67 expression did not differ by PR expression.

**Table 1:** Baseline characteristics.

	High PR (*N* = 202)	Low PR (*N* = 70)	*P*-Value^a^
Median age, years (range)	57 (31–91)	49 (26–81)	<0.001^b^
T stage			0.139
T1	113 (55.9)	32 (45.7)	
T2	89 (44.1)	38 (54.3)	
N stage			0.194^c^
N0	124 (61.4)	51 (72.9)	
N1	66 (32.7)	14 (20.0)	
N2	8 (4.0)	4 (5.7)	
N3	4 (2.0)	1 (1.4)	
Stage			0.751
I	79 (39.1)	24 (34.3)	
II	108 (53.5)	41 (58.6)	
III	15 (7.4)	5 (7.1)	
Histologic grade^d^			0.064
I	60 (30.3)	15 (22.4)	
II	109 (55.1)	34 (50.7)	
III	29 (14.6)	18 (26.9)	
Estrogen receptor^e^			0.019
High	184 (91.1)	57 (81.4)	
Low	18 (8.9)	13 (18.6)	
Ki67			0.980
≥20%	43 (21.3)	15 (21.4)	
<20%	159 (78.7)	55 (78.6)	

^a^Chi-square test except; ^b^Mann–Whitney *U* test; ^c^Fisher exact test; ^d^missing values; ^e^high, Allred score 5–8; low, Allred score 0–4.

### High *TP53* mutation rates in low-PR tumors

**Figure 1:**
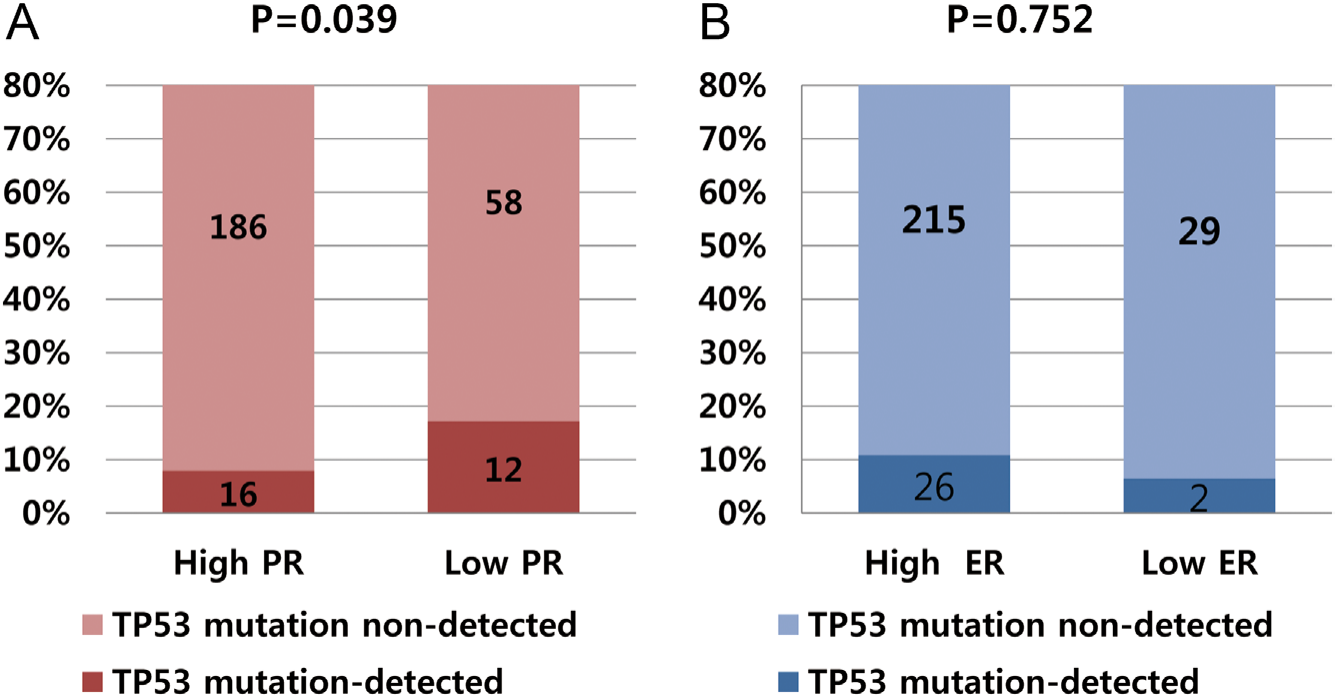
*TP53* mutation rates according to PR and ER status. (A) The *TP53* mutation rate was significantly higher in low-PR tumors than that in high-PR tumors (17.1% vs 7.9%, *P* = 0.039). (B) The rates were not statistically different according to ER expression (10.8% in high-ER tumors vs 6.5% in low ER-tumors, *P* = 0.752).

Tumors from 28 patients (10.3%) had a somatic *TP53* mutation. The diverse locations of mutations within the *TP53* coding sequence are shown in Supplementary data 1 (see section on supplementary data given at the end of this article). Among six ‘hotspot’ residues (R175, G245, R248, R249, R273 and R282) (Cho* et al*. 1994), our patients had two R248 and two R273 missense mutations. Missense mutations were most common (19 of 28; 67.9%), followed non-sense mutations (4 of 28), splicing mutations (3 of 28) and frame-shift mutations (2 of 28) (Supplementary data 1). The *TP53* mutation rate was significantly higher in low-PR tumors than that in high-PR tumors (17.1% vs 7.9%, *P* = 0.039; Fig. 1A). However, the *TP53* mutation rate was not statis­tically different according to ER expression (6.5% in low-ER-tumors vs 10.8% in high-ER tumors, *P* = 0.752; Fig. 1B).

### SUV in relation to PR expression

**Figure 2:**
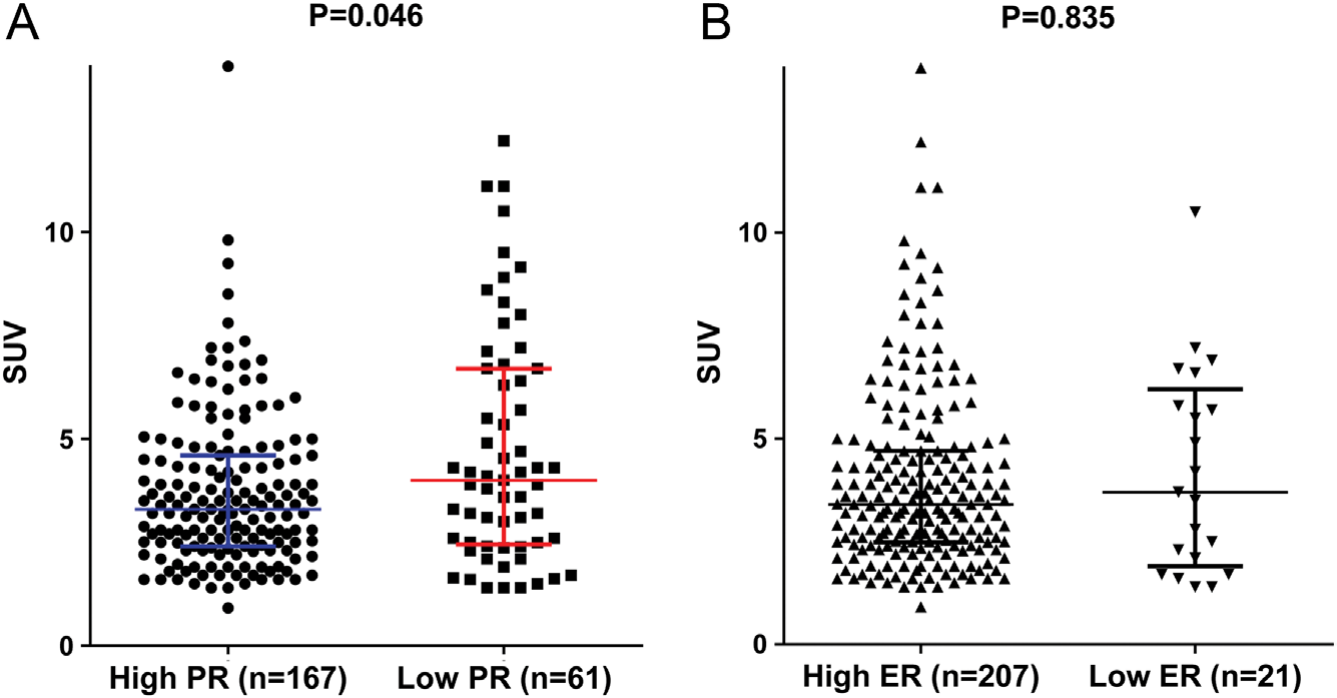
Distribution of SUVs according to PR and ER status. (A) Low-PR tumors had a significantly higher median SUV than that of high-PR tumors (*P* = 0.046, Mann–Whitney *U* test). The median SUVs for low- and high-PR tumors were 4 (1.4–12.2) and 3.3 (0.91–14), respectively. (B) The median SUVs were not statistically different according to ER expression (*P* = 0.835, Mann–Whitney *U* test). The median SUVs for low- and high-ER tumors were 3.7 (1.4–10.5) and 3.4 (0.91–14.0), respectively.

To investigate the pathologic characteristics of tumors in terms of glucose uptake, we measured SUV in patients undergoing preoperative 18f-FDG-PET. We obtained SUVs from 229 patients (84.2%). We compared the SUV distributions according to PR expression because the SUVs were not normally distributed (*P* < 0.001, Kolmogorov–Smirnov test). The SUV distributions differed significantly according to PR expression (*P* = 0.046, Mann–Whitney *U* test), indicating that low-PR tumors had a significantly higher median SUV than that of high-PR tumors (Fig. 2A). However, the median SUVs were not statistically different according to ER expression (*P* = 0.835, Fig. 2B).

### SUV is predictive of low PR expression

We identified the factors associated with low-PR expression using binary logistic regression analysis. The variables with *P* < 0.05 in univariate analysis included age, *TP53* mutation, tumor grade and SUV (Table 2). Multivariable analysis revealed that SUV and age remained independent variables associated with low PR expression (Table 2). Continuous SUV had an odds ratio (OR = 1.220; 95% confidence interval (CI) = 1.068–1.394) for predicting low-PR tumors in multivariable analysis.

**Table 2:** Binary logistic regression analysis to identify predictive factors for low PR expression.

Variables	Univariable		Multivariable	
	*P*-Value	OR (95% CI)	*P*-Value	OR (95% CI)
Age (per 1 year)	0.001	1.044 (1.018–1.071)	0.003	1.043 (1.014–1.073)
*TP53* mutation	0.033	2.405 (1.076–5.377)		
Grade				
I		Ref		
II	0.526	1.248 (0.629–2.474)		
III	0.029	2.483 (1.098–5.615)		
SUV (per 1 unit)	0.003	1.218 (1.070–1.387)	0.003	1.220 (1.068–1.394)
T stage				
T1		Ref		
T2	0.141	1.508 (0.873–2.603)		
N stage				
N0		Ref		
N1	0.050	0.516 (0.266–1.000)		
N2	0.758	1.216 (0.350–4.217)		
N3	0.660	0.608 (0.066–5.571)		
Stage				
I		Ref		
II	0.453	1.250 (0.699–2.235)		
III	0.870	1.097 (0.361–3.331)		
ER expression^a^				
High		Ref		
Low	0.021	2.351 (1.135–4.868)		
Ki67				
≥20%		Ref		
<20%	0.98	0.992 (0.511–1.924)		

^a^High, Allred score 5–8; low, Allred score 0–4.

ER, estrogen receptor; SUV, standardized uptake.

### Survival analysis

At a median follow-up of 50 months (3–134), 18 patients experienced tumor recurrence, including 17 distant metastases and one loco-regional recurrence. Among the 18 patients with recurrence, three had both metastases and loco-regional recurrences. During the follow-up period, one patient died from breast cancer, and one patient died from a non-cancerous cause. The adjuvant treatments, presented online, were not statistically different according to PR expression (Supplementary data 2).

**Figure 3:**
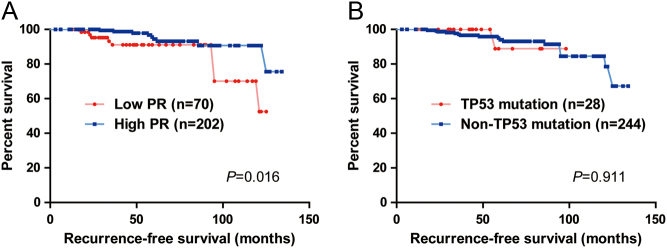
Kaplan–Meier plots of recurrence-free survival (RFS). (A) The RFS differed significantly according to PR expression (*P* = 0.016, log-rank test). (B) However, the RFS did not differ significantly according to the presence of *TP53* mutation (*P* = 0.911, log-rank test).

The Kaplan–Meier plots for RFS differed significantly according to PR expression (*P* = 0.016; Fig. 3A). The RFS was longer in the groups with high-PR tumors; however, a significant difference of the RFS according to the presence of *TP53* mutation was not observed (*P* = 0.911; Fig. 3B).

In the univariable analyses using the Cox regression model, T stage, N stage, stage, SUV and PR expression were significant (Table 3). In the multivariable model, stage and PR expression were significant prognostic factors for the RFS (Table 3). Then, we compared a predictive ability of the multivariable model with or without SUV using the Harrell c-statistics. For the model 1 (without SUV), the Harrell c-index was 0.713. For the model 2 (with SUV), the Harrell c-index was 0.687, indicating that SUV does not add a predictive power to the model 1 that includes stage and PR expression.

**Table 3:** A cox-regression hazard model for recurrence-free survival.

Variables	Univariable		Multivariable			
	*P*-Value	HR (95% CI)	Model 1 (backward)		Model 2 (backward + SUV)	
			*P*-Value	HR (95% CI)	*P*-Value	HR (95% CI)
Age	0.740	1.008 (0.963–1.054)				
*TTP53* mutation	0.912	0.891 (0.116–6.848)				
SUV	0.046	1.231 (1.004–1.509)			0.577	1.071 (0.842–1.362)
Grade						
I	0.482	Ref				
II	0.381	1.528 (0.468–4.987)				
III	0.046	1.970 (0.432–8.985)				
T stage						
T1		Ref				
T2	0.004	5.235 (1.674–16.372)				
N stage						
N0		Ref				
N1	0.376	0.562 (0.157–2.010)				
N2	0.700	1.497 (0.192–11.709)				
N3	0.000	18.300 (3.873–86.470)				
Stage						
I		Ref		Ref		Ref
II	0.080	3.190 (0.872–11.661)	0.079	3.208 (0.872–11.795)	0.245	2.237 (0.576–8.684)
III	0.007	8.031 (1.791–36.015)	0.007	7.980 (1.776–35.852)	0.061	7.164 (0.911–56.332)
ER expression						
High		Ref				
Low	0.473	0.580 (0.131–2.572)				
Ki67						
≥20%		Ref				
<20%	0.175	0.506 (0.189–1.355)				
PR expression			0.024		0.067	
High		Ref		Ref		Ref
Low	0.023	2.955 (1.165–7.499)		2.953 (1.152–7.567)		2.793 (0.932–8.367)
Harrell c-index			0.718		0.687	

ER, estrogen receptor; PR, progesterone receptor; SUV, standardized uptake value.

## Discussion

In this study, tumors with low PR expression more frequently had *TP53* mutations and elevated glucose uptake compared to those in tumors with high PR expression, providing a biological rationale for why low PR expression could be a biomarker to distinguish aggressive tumors from ER-positive/HER2-negative tumors, similar to high Ki67 level or poor histologic grade. Our findings are in line with those of previous studies indicating that the loss of PR expression correlates with aggressive tumor characteristics (Arpino *et al*. 2005, Olivier *et al*. 2006) and with a gene signature of the PI3K/Akt/mTOR pathway (Creighton *et al*. 2009).

An earlier study of 1794 breast cancer patients showed that *TP53* mutations were more frequent in PR-negative tumors than those in PR-positive tumors (Olivier *et al*. 2006) but did not identify HER2 status, remaining unclear whether the *TP53* mutation rate is higher in PR-lacking tumors within ER-positive/HER2-negative tumors. We observed that the rate was significantly higher in low-PR tumors within luminal/HER2-negative tumors, suggesting that frequent *TP53* pathway defects could characterize ER-positive/PR-low tumors even without HER2 amplification.

A comprehensive genomic study using whole genome analyses showed that *TP53* mutations contribute to endocrine failure in luminal breast cancer (Ellis* et al*. 2012). A bioinformatics analysis integrating gene copy number aberrations, gene expression profiles and mutations indicated that *TP53* mutations play an integral role in determining luminal B status and resistance to endocrine therapy (Ellis *et al*. 2012). Furthermore, ~30% of luminal B tumors harbored *TP53* mutant in TCGA data (Cancer Genome Atlas Network 2012), suggesting that a considerable number of luminal B tumors have an impaired *TP53* pathway. Our observations of *TP53* mutations and low PR expression consolidate a current consensus that low PR expression can be used to identify luminal B-like tumors (Goldhirsch *et al*. 2013, Coates* et al*. 2015) and provide a rationale that low-PR tumors have a worse prognosis than high-PR tumors even though they are all ER-positive/HER2-negative tumors.

In addition to *TP53* mutations, we also characterized the low-PR tumors using the SUV as an objective numerical value indicative of glucose uptake within tumors. Based on a study elucidating the tumor biology of increasing SUV such as MYC-overexpression (Palaskas* et al*. 2011) or centromere protein F-expression, breast cancer with high SUV levels could indicate aggressive tumors. Moreover, our previous study showed that high SUV could be a poor prognostic factor in luminal breast cancer, also concordant with another study (Aogi* et al*. 2015), which similarly evaluated the prognosis of luminal breast cancer according to SUV. In another study of luminal/HER2-negative tumors, we found that SUV is correlated with the 21-gene recurrence score and inversely correlated with PR mRNA expression (Ahn *et al*. 2017). Our previous finding on the inverse correlation between SUV and PR mRNA expression is concordant with the findings of this study that low-PR tumors had a higher SUV. Several lines of evidence suggest that growth factor signaling is frequently activated in PR-lacking tumors (Cui* et al*. 2005), supporting our findings that enhanced growth factor pathway expression may contribute to increased glycolysis (Vander Heiden* et al*. 2009). Further studies on the relationship between the loss of PR and increased glycolytic activity in this subset of breast cancer are warranted.

Our major limitation is the potential argument against the optimal PR expression threshold. Although Prat *et al*. suggested a cut-off of 20% (Prat *et al*. 2013) based on the relationship between mRNA levels of the PR gene and IHC examination, it is still hard to clearly distinguish low and high PR tumors using IHC examination alone. Expert panels largely agreed that low PR expression can be utilized as a determinant for luminal B-like tumors (Goldhirsch *et al*. 2013, Coates *et al*. 2015), but recommended its combination with other pathologic markers such as Ki67 and grade (Coates *et al*. 2015). More research is needed to determine the optimal PR expression cut-off.

Another limitation is noted in the survival analyses. In multivariable model 1, the poor prognostic impact of low PR expression was observed but disappeared in multivariable model 2. Also, the negative impact of high SUV on survival suggested by previous studies was not found in model 2. This might be due to the small number of recurrences and missing SUV data. Much longer follow-up is required to identify the influence of PR status and SUV in those patients because luminal/HER2-negative patients generally have a very favorable outcome. In addition, a method of selective hot-spot sequencing could be regarded as a limitation because it does not discover any kinds of genetic change of whole exome. Also, high SUV in low PR-tumors cannot be excluded as a mere reflection of poorly differentiated carcinoma because low-PR tumors tend to have high histologic grade.

Despite these limitations, our study characterized low-PR tumors in terms of altered *TP53* pathway and glucose metabolism. It is worth exploring low-PR tumors in viewpoints of these biologic pathways. Moreover, these characteristics of low-PR tumors partly explain why low-PR tumors have a worse outcome among luminal/HER2-negative breast cancer that generally shows a favorable course.

## Conclusions

We provide evidence that genetic alteration of the *TP53* gene and dysregulated glucose metabolism partly involve low PR expression in ER-positive and HER2-negative breast cancer.

## Supplementary Material

Supporting Figure 1

Supplementary Table 1

## Declaration of interest

The authors declare that there is no conflict of interest that could be perceived as prejudicing the impartiality of the research reported.

## Funding

This research was supported by the Basic Science Research Program through the NRF, funded by the Ministry of Science, ICT, & Future Planning (NRF-2015R1C1A1A02037104 and NRF-2016R1D1A1A09917675); a grant from the National R&D Program for Cancer Control, Ministry of Health & Welfare, Republic of Korea (1520120); and a new faculty research seed money grant from Yonsei University College of Medicine (2016-32-0025).

## Availability of data and materials

The datasets generated and analyzed during the current study are available from the corresponding author on request.

## Ethics approval and consent to participate

The study protocol was reviewed and approved by the Institutional Review Boards of Gangnam Severance Hospital, Yonsei University, Seoul, Korea, and adhered to the tenets of the Declaration of Helsinki. Owing to the retrospective approach of this study, the need for informed consent was waived by the ethics committees.

## References

[bib3] Ahn SG Lee M Jeon TJ Han K Lee HM Lee SA Ryu YH Son EJ Jeong J 2014a [18F]-fluorodeoxyglucose positron emission tomography can contribute to discriminate patients with poor prognosis in hormone receptor-positive breast cancer. PLoS ONE 9 e105905 (10.1371/journal.pone.0105905) 25167062 PMC4148332

[bib4] Ahn SG Park JT Lee HM Lee HW Jeon TJ Han K Lee SA Dong SM Ryu YH Son EJ, ***et al*** 2014b Standardized uptake value of (1)(8)F-fluorodeoxyglucose positron emission tomography for prediction of tumor recurrence in breast cancer beyond tumor burden. Breast Cancer Research 16 502 (10.1186/s13058-014-0502-y) 25551703 PMC4308858

[bib2] Ahn SG Lee JH Lee HW Jeon TJ Ryu YH Kim KM Sohn J Yun M Lee SA Jeong J, ***et al*** 2017 Comparison of standardized uptake value of 18F-FDG-PET-CT with 21-gene recurrence score in estrogen receptor-positive, HER2-negative breast cancer. PLoS ONE 12 e0175048 (10.1371/journal.pone.0175048) 28419166 PMC5395149

[bib5] Aogi K Kadoya T Sugawara Y Kiyoto S Shigematsu H Masumoto N Okada M 2015 Utility of (18)F FDG-PET/CT for predicting prognosis of luminal-type breast cancer. Breast Cancer Research and Treatment 150 209–217. (10.1007/s10549-015-3303-9) 25697596 PMC4344554

[bib6] Arpino G Weiss H Lee AV Schiff R De Placido S Osborne CK Elledge RM 2005 Estrogen receptor-positive, progesterone receptor-negative breast cancer: association with growth factor receptor expression and tamoxifen resistance. Journal of the National Cancer Institute 97 1254–1261. (10.1093/jnci/dji249) 16145046

[bib7] Bertheau P Lehmann-Che J Varna M Dumay A Poirot B Porcher R Turpin E Plassa LF de Roquancourt A Bourstyn E, ***et al*** 2013 p53 in breast cancer subtypes and new insights into response to chemotherapy. Breast 22 (Supplement 2) S27–S29. (10.1016/j.breast.2013.07.005) 24074787

[bib8] Cancello G Maisonneuve P Rotmensz N Viale G Mastropasqua MG Pruneri G Montagna E Iorfida M Mazza M Balduzzi A, ***et al*** 2013 Progesterone receptor loss identifies luminal B breast cancer subgroups at higher risk of relapse. Annals of Oncology 24 661–668. (10.1093/annonc/mds430) 23022996

[bib1] Cancer Genome Atlas Network 2012 Comprehensive molecular portraits of human breast tumours. Nature 490 61–70. (10.1038/nature11412) 23000897 PMC3465532

[bib9] Cho Y Gorina S Jeffrey PD Pavletich NP 1994 Crystal structure of a p53 tumor suppressor-DNA complex: understanding tumorigenic mutations. Science 265 346–355. (10.1126/science.8023157) 8023157

[bib10] Clark GM McGuire WL Hubay CA Pearson OH Marshall JS 1983 Progesterone receptors as a prognostic factor in Stage II breast cancer. New England Journal of Medicine 309 1343–1347. (10.1056/NEJM198312013092240) 6633596

[bib11] Coates AS Winer EP Goldhirsch A Gelber RD Gnant M Piccart-Gebhart M Thurlimann B Senn HJ 2015 Tailoring therapies – improving the management of early breast cancer: St Gallen International Expert Consensus on the Primary Therapy of Early Breast Cancer 2015. Annals of Oncology 26 1533–1546. (10.1093/annonc/mdv221) 25939896 PMC4511219

[bib12] Creighton CJ Kent Osborne C van de Vijver MJ Foekens JA Klijn JG Horlings HM Nuyten D Wang Y Zhang Y Chamness GC, ***et al*** 2009 Molecular profiles of progesterone receptor loss in human breast tumors. Breast Cancer Research and Treatment 114 287–299. (10.1007/s10549-008-0017-2) 18425577 PMC2635926

[bib13] Cui X Schiff R Arpino G Osborne CK Lee AV 2005 Biology of progesterone receptor loss in breast cancer and its implications for endocrine therapy. Journal of Clinical Oncology 23 7721–7735. (10.1200/jco.2005.09.004) 16234531

[bib14] Desmedt C Voet T Sotiriou C Campbell PJ 2012 Next-generation sequencing in breast cancer: first take home messages. Current Opinion in Oncology 24 597–604. (10.1097/CCO.0b013e328359554e) 23014189 PMC3713550

[bib15] Edge SB Compton CC 2010 The American Joint Committee on Cancer: the 7th edition of the AJCC cancer staging manual and the future of TNM. Annals of Surgical Oncology 17 1471–1474. (10.1245/s10434-010-0985-4) 20180029

[bib16] Ellis MJ Ding L Shen D Luo J Suman VJ Wallis JW Van Tine BA Hoog J Goiffon RJ Goldstein TC, ***et al*** 2012 Whole-genome analysis informs breast cancer response to aromatase inhibition. Nature 486 353–360. (10.1038/nature11143) 22722193 PMC3383766

[bib17] Goldhirsch A Winer EP Coates AS Gelber RD Piccart-Gebhart M Thurlimann B Senn HJ 2013 Personalizing the treatment of women with early breast cancer: highlights of the St Gallen International Expert Consensus on the Primary Therapy of Early Breast Cancer 2013. Annals of Oncology 24 2206–2223. (10.1093/annonc/mdt303) 23917950 PMC3755334

[bib18] Harrell FE Jr Lee KL Mark DB 1996 Multivariable prognostic models: issues in developing models, evaluating assumptions and adequacy, and measuring and reducing errors. Statistics in Medicine 15 361–387. (10.1002/(sici)1097-0258(19960229)15:4<361::Aid-sim168>3.0.Co;2-4) 8668867

[bib19] Harvey JM Clark GM Osborne CK Allred DC 1999 Estrogen receptor status by immunohistochemistry is superior to the ligand-binding assay for predicting response to adjuvant endocrine therapy in breast cancer. Journal of Clinical Oncology 17 1474–1481. (10.1200/jco.1999.17.5.1474) 10334533

[bib20] Horwitz KB McGuire WL 1975 Predicting response to endocrine therapy in human breast cancer: a hypothesis. Science 189 726–727. (10.1126/science.168640) 168640

[bib22] Kim Y Kim J Lee HD Jeong J Lee W Lee KA 2013 Spectrum of EGFR gene copy number changes and KRAS gene mutation status in Korean triple negative breast cancer patients. PLoS ONE 8 e79014 (10.1371/journal.pone.0079014) 24205362 PMC3813621

[bib21] Kim HW Lee HM Hwang SH Ahn SG Lee KA Jeong J 2014 Patterns and biologic features of p53 mutation types in korean breast cancer patients. Journal of Breast Cancer 17 1–7. (10.4048/jbc.2014.17.1.1) 24744791 PMC3988337

[bib23] Lee HW Lee HM Choi SE Yoo H Ahn SG Lee MK Jeong J Jung WH 2016 The prognostic impact of early change in 18F-FDG PET SUV after neoadjuvant chemotherapy in patients with locally advanced breast cancer. Journal of Nuclear Medicine 57 1183–1188. (10.2967/jnumed.115.166322) 27033896

[bib24] Olivier M Langerod A Carrieri P Bergh J Klaar S Eyfjord J Theillet C Rodriguez C Lidereau R Bieche I, ***et al*** 2006 The clinical value of somatic TP53 gene mutations in 1,794 patients with breast cancer. Clinical Cancer Research 12 1157–1167. (10.1158/1078-0432.Ccr-05-1029) 16489069

[bib25] Palaskas N Larson SM Schultz N Komisopoulou E Wong J Rohle D Campos C Yannuzzi N Osborne JR Linkov I, ***et al*** 2011 18F-fluorodeoxy-glucose positron emission tomography marks MYC-overexpressing human basal-like breast cancers. Cancer Research 71 5164–5174. (10.1158/0008-5472.can-10-4633) 21646475 PMC3148325

[bib26] Prat A Cheang MC Martin M Parker JS Carrasco E Caballero R Tyldesley S Gelmon K Bernard PS Nielsen TO, ***et al*** 2013 Prognostic significance of progesterone receptor-positive tumor cells within immunohistochemically defined luminal A breast cancer. Journal of Clinical Oncology 31 203–209. (10.1200/jco.2012.43.4134) 23233704 PMC3532392

[bib27] Vander Heiden MG Cantley LC Thompson CB 2009 Understanding the Warburg effect: the metabolic requirements of cell proliferation. Science 324 1029–1033. (10.1126/science.1160809) 19460998 PMC2849637

[bib28] Wolff AC Hammond ME Hicks DG Dowsett M McShane LM Allison KH Allred DC Bartlett JM Bilous M Fitzgibbons P, ***et al*** 2013 Recommendations for human epidermal growth factor receptor 2 testing in breast cancer: American Society of Clinical Oncology/College of American Pathologists clinical practice guideline update. Journal of Clinical Oncology 31 3997–4013. (10.1200/jco.2013.50.9984) 24101045

