# Supplementary figures and images for: Low PR in ER(+)/HER2(−) breast cancer: high rates of TP53 mutation and high SUV
Source: Endocr Relat Cancer. 2018 Nov 8;26(2):177–85. doi: 10.1530/ERC-18-0281 (PMC6347277; doi:10.1530/ERC-18-0281)

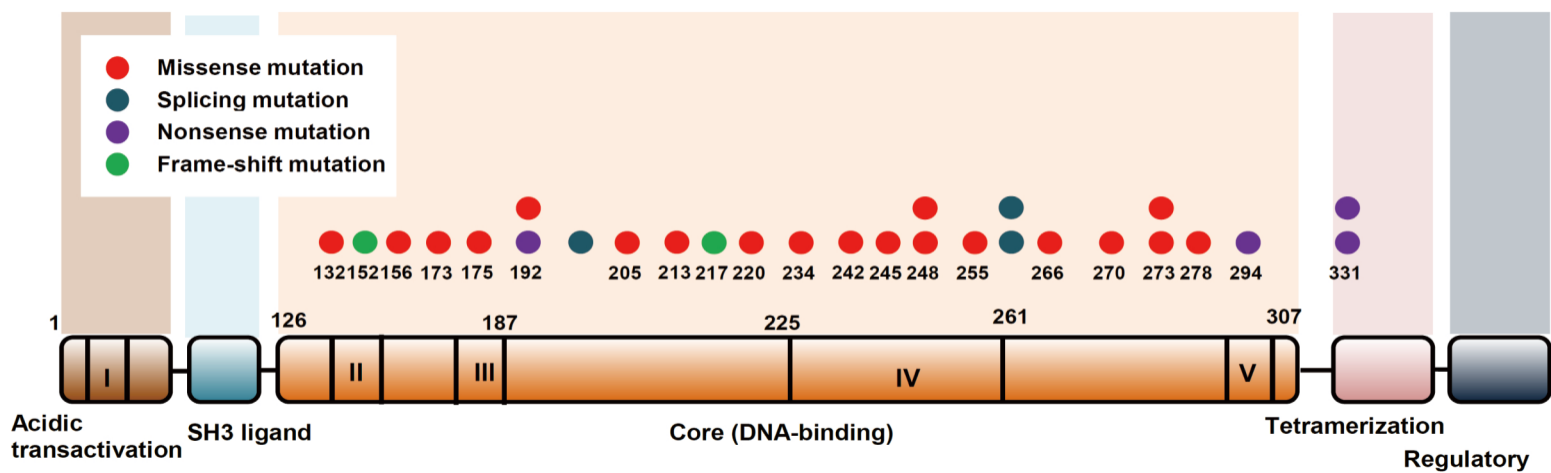

Supplement: Supporting Figure 1 [file supplementary_figure_1.pdf]
