# Supplementary material for: Low PR in ER(+)/HER2(−) breast cancer: high rates of TP53 mutation and high SUV
Source: Endocr Relat Cancer. 2018 Nov 8;26(2):177–85. doi: 10.1530/ERC-18-0281 (PMC6347277; doi:10.1530/ERC-18-0281)
Supplement: Supplementary Table 1 [file supplementary_table_1.pdf]

Supplementary File 2. Adjuvant treatments

	High PR (N = 202)	Low PR (N = 70)	P-value
**Chemotherapy**			0.781
Administered	110 (54.5)	40 (57.1)	
Not administered	92 (45.5)	30 (42.9)	
**Radiotherapy**			0.781
Administered	93 (46.0)	34 (48.6)	
Not administered	109 (54.0)	36 (51.4)	
